# Socio-economic, Knowledge Attitude Practices (KAP), household related and demographic based appearance of non-dengue infected individuals in high dengue risk areas of Kandy District, Sri Lanka

**DOI:** 10.1186/s12879-018-2995-y

**Published:** 2018-02-21

**Authors:** Lahiru Udayanga, Nayana Gunathilaka, M. C. M. Iqbal, Kusumawathie Pahalagedara, Upali S. Amarasinghe, Wimaladharma Abeyewickreme

**Affiliations:** 1 Molecular Medicine Unit, Faculty of Medicine, University of Kelaniya, Ragama, Sri Lanka; 2 Deparrment Parasitology, Faculty of Medicine, University of Kelaniya, Ragama, Sri Lanka; 3 National Institute of Fundamental Studies, Kandy, Sri Lanka; 4 Anti Malaria Campaign, Regional Office-Kandy, Kandy, Sri Lanka; 5 Department of Zoology and Environment Management, Faculty of Science, University of Kelaniya, Kelaniya, Sri Lanka; 6 Department of Paraclinical Science, Faculty of Medicine, Sir John Kotelawala Defense University, Ratmalana, Sri Lanka

**Keywords:** Dengue, Knowledge attitude practices, Sri Lanka

## Abstract

**Background:**

Socio-economic, demographic factors and Knowledge Attitude Practices (KAPs) have been recognized as critical factors that influence the incidence and transmission of dengue epidemics. However, studies that characterize above features of a risk free or low risk population are rare. Therefore, the present study was conducted to characterize the household related, demographic, socio-economic factors and KAPs status of five selected dengue free communities.

**Method:**

An analytical cross-sectional survey was conducted on selected demographic, socio-economic, household related and KAPs in five selected dengue free communities living in dengue risk areas within Kandy District, Central Province, Sri Lanka. Household heads of 1000 randomly selected houses were interviewed in this study. Chi-square test for independence, cluster analysis and Principal Coordinates (PCO) analysis were used for data analysis.

**Results:**

Knowledge and awareness regarding dengue, (prevention of the vector breeding, bites of mosquitoes, disease symptoms and waste management) and attitudes of the community (towards home gardening, composting, waste management and maintenance of a clean and dengue free environment) are associated with the dengue free status of the study populations.

**Conclusions:**

The vector controlling authorities should focus on socio-economic, demographic and KAPs in stimulating the community to cooperate in the integrated vector management strategies to improve vector control and reduce transmission of dengue within Kandy District.

**Electronic supplementary material:**

The online version of this article (10.1186/s12879-018-2995-y) contains supplementary material, which is available to authorized users.

## Background

Dengue fever (DF), transmitted by the bites of *Aedes aegypti* (primary vector) and *Aedes albopictus* (secondary vector) mosquitoes, is a viral infection that is now considered as the world’s fastest growing vector borne disease [1]. Four serotypes of dengue virus (DENV-1, 2, 3, and 4) have been identified as the cause of dengue, which are capable of resulting in an array of progressive severe conditions ranging from least severe DF to Dengue Haemorrhagic Fever (DHF) and the most severe condition, Dengue Shock Syndrome (DSS) [1]. In the recent past, dengue has drastically expanded over a significant geographic range in the world [2]. At the global scale, approximately 3.9 billion people residing in 128 countries, are recognized to be at risk from dengue infections, emphasizing the severity of dengue [2]. Rapid expansions in urbanization, increasing world population, international trade/travel and inadequate methods of control, are some of the key factors responsible for the increasing rate of dengue in the past few decades, extending into new territories and more rural areas in many countries [1–3].

In Sri Lanka since 1989, dengue is recognized as a regular epidemic, indicating an exponential increase in the incidence [4, 5]. There are significant spatial and temporal trends in the emergence of dengue outbreaks in Sri Lanka, which could be mainly accounted by the variations in environmental, meteorological, and socio-economic factors throughout the country [5]. In 2016, 54,945 of suspected dengue cases have been reported in Sri Lanka, followed by 184, 442 cases within 2017, indicating the severity of the issue [6]. The large annual financial allocation for the management of dengue patients and controlling of dengue vectors, has made dengue to be recognized as a priority health issue in Sri Lanka that imposes a heavy burden on the national health budget.

The incidence and spread of dengue is influenced by a variety of factors such as socio-economic conditions, degree and intensity of imposed management/control actions, socio-cultural practices, environmental factors, and changes in the climate [7]. However, a holistic approach considering these aspects has rarely been documented or studied in Sri Lanka, to the best of our knowledge.

Socio-economic, demographic and Knowledge Attitude Practices (KAPs) are considered as critical factors that influence the incidence and transmission of dengue. Notwithstanding the efforts to develop an effective vaccine for all four serotypes of the virus, the only practical solution for controlling dengue is the reduction of the vector population. For this, the effective participation of the local communities is essential at the ground level [8–9]. Despite the awareness campaigns conducted via conventional and social media or at the ground level, their effectiveness and the extent to which the awareness of people have increased, still remain unclear within Sri Lanka. Further, the extent to which practical implementation of the knowledge on control measures provided to the community, also remains to be less investigated. Hence, KAPs of the people in Sri Lanka, especially on dengue vectors, their breeding, disease transmission and vector management should be systematically analyzed and further improved to identify gaps in current vector controlling activities in order to establish effective community based vector controlling programmes.

Studies that characterize the features of a risk free or low risk population (which are often limited or none), can reveal KAPs that are effective and time tested that can be adopted by high risk communities since they may share the same social environment. Therefore, studies of this nature are essential since they enable the comparative characterization of risk factors among high and low risk populations, in order to adopt appropriate regulations or approaches based on the social and micro environment [9]. Hence, the present study was conceived and conducted in this context to characterize the demographic, household related, socio-economic and KAPs status of five selected dengue free communities living in dengue risk areas, within the District of Kandy in Sri Lanka.

## Method

### Study area

**Fig. 1:**
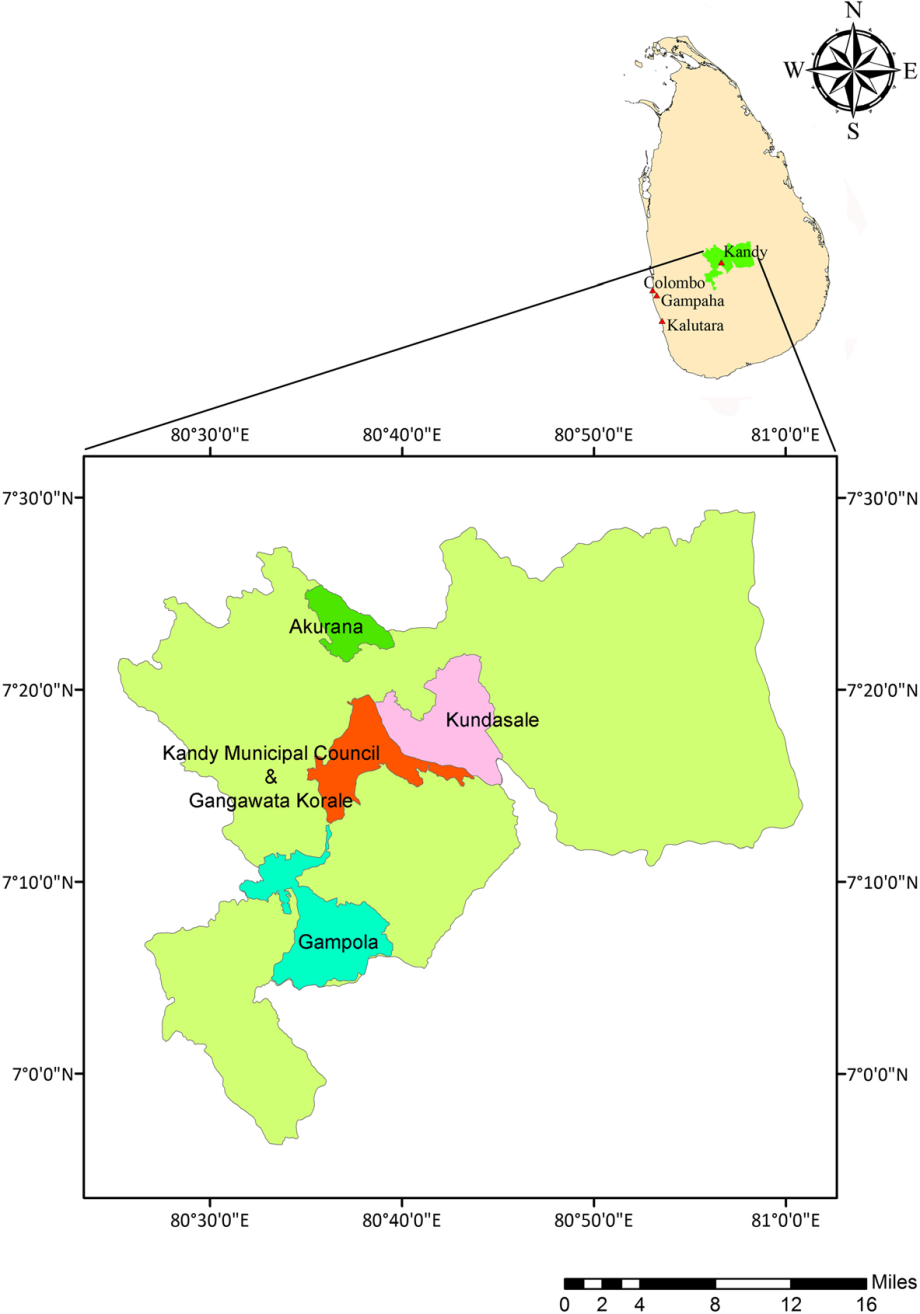
Map of Kandy District showing study areas [Kandy Municipal Council (KMC), Gangawata Korale (GK), Kundasale and Gampola, and Akurana] along with the geographical location of Kandy District in Sri Lanka

The Kandy District Secretariat Division (DSD) is located in the Kandy plateau (69.56° to 70.29°N and 80.25° to 80.00°E), and consists of 20 regional local government institutions, extending over an area of 1940 km^2^. The study area hosts a multi-culture and multi-ethnic population of 1,369,899 with a population density of 710 km^− 2^ as suggested by the Census data in 2012 [10]. The study area remains as one of the major tourist interests due to its natural location, historical and religiously important places. At present, Kandy District remain as the third highest risk area for dengue transmission in the country contributing to 7.77% (14, 338) of the total dengue cases reported during the year [6]. Among the 23 Medical Officer of Health (MOH) areas in the Kandy District, five MOH areas, namely Kandy Municipal Council area (KMC), Gampola, Akurana, Kundasale and Gangawata Korale (GK), which recorded the highest number of dengue cases during 2010–2015 were selected as study areas (Fig. 1).

### Selection of study population

The analytical cross-sectional survey was conducted from April to November, 2016. A total of 15 Grama Niladhari Divisions (the local level administrative units; GNDs) were identified from 5 selected MOH areas (3 each) that have reported the highest number of dengue cases during 2010–2015. A sample of 1000 households from the five selected MOH areas (200 households from each MOH area; 200 × 5) was selected on a random basis following Krejcie and Morgan [11]. During the calculation of sample size, it was assumed that the marginal error is 3.5%, population proportion is 0.5, while the actual population size of the whole Kandy district is 1, 369, 899.

The houses listed in the residence registries of the households in selected 15 GNDs were numbered systematically and households were selected randomly using a three-digit random number table for the present study. Households of which the residents were not willing to cooperate in the study due to one or more reasons such as religious beliefs, absence of a household head or an opinion that it is not worth participating, were not considered for the survey. In such occasions, the sample size was achieved by selecting new households (instead of the rejected), following the above procedure.

### Data collection

An interviewer-administered questionnaire prepared in the three local languages (Sinhala, English and Tamil), was used to obtain the demographic, socio-economic, household related and Knowledge, Attitude and Practices (KAPs) information of the selected households in each MOH area (Additional file 1). The selected household head was interviewed by a group of trained interviewers. The household head was defined as the person who is perceived by the household members to be the primary decision maker in the family and the household was defined as individuals living together and taking meals from a common cooking facility [12]. In the absence of a household head, a responsible adult above 18 years, appointed by the family, was considered for the study. The data were organized under 4 major aspects as described below.

#### Personal and socio-demographic characteristics

Age and sex of the household head, number of family members, monthly income of the family and number of residing years within the relevant MOH were collected.

#### Household characteristics

The size of the homestead, vegetation coverage, nature of the premises, accessibility conditions, surrounding landuse types, number of rooms in the house, roofing, drinking water source and sanitary conditions of the households were examined*.*

#### Knowledge attitude and practices

##### Knowledge

Transmission of dengue, vector biology, clinical symptoms, responsibilities of the community to control vector breeding, names of major dengue vectors, morphological features and life cycle, breeding habitats, active time of dengue vectors, treatments for dengue if any, preventive measures against dengue and role of the community participation in dengue prevention and control were assessed using an oral interviewer-administered questionnaire comprised of 12 questions. The levels of awareness of the participants were classified in to three categories as “Good” (> 66.67%), “Moderate” (33.34 to 66.66%) and “Poor” (< 33.33%) based on a percentage score obtained for the above questionnaire.

##### Attitudes

Importance of maintaining a clean and vector breeding free environment, source separation and proper Solid Waste Management (SWM), knowledge deficiency on dengue in relevance to symptoms, patient care, vector control, SWM and the willingness to take part in community based vector control programmes.

##### Practices

Source separation of solid waste, waste disposal methods, maintaining a clean environment, home gardening practices and composting, covering of water storage tanks, cleaning and maintenance of roof gutters, use of mosquito repellents and insecticides.

#### Role of vector control entities (VCE)

Condition of the previously implemented vector controlling programmes, active Vector Control Entities (VCE) within the study area, nature of the service provided by the Public Health Inspector (PHI) in the relevant MOH area.

### Data interpretation and statistical analysis

All the collected data were encoded into Microsoft Access® data sheets (Version, 2007), while adhering to quality controlling procedures. The accuracy of data were routinely checked by cross tabulations and logical checks. Discrepant data were checked against original data forms and if any mistakes were promptly corrected.

Epidemiological, demographic, socio-economic and KAP factors that characterize the low dengue risk transmission were statistically compared for homogeneity of proportions using the chi-square test for Independence [13, 14]. Partitioning of the contingency tables to enable the comparison at two study population level by using the chi-square test for Independence, was followed as a post-hoc test for parameters with statistical significance at 5% level of significance. The proportions of demographic, epidemiological and socio-economic characteristics of the five populations studied, were square-root transformed and a cluster analysis (with respect to Euclidean distance [15]) followed by Analysis of Similarities (ANOSIM) (i.e., a nonparametric analog of MANOVA) [16]. Principal Coordinates (PCO) analysis was also performed to highlight and visually represent the underlying segregation patterns of the study populations based on multivariate data sets of demographic, epidemiological and socio-economic characteristics of the five populations studied. The Plymouth Routines in Multivariate Ecological Research version 6 (PRIMER 6) was used to perform the statistical comparisons [15]. Further, identified factors that would be responsible for low dengue risk transmission, were compared with recognized risk factors as in the published literature.

## Results

### Demographic factors

A total of 1000 households were interviewed. The age group of 35–55 years was predominant among the study populations, while the age group of 15–35 years was the lowest, except in Gampola (Table 1). The average family size was 4–6 individuals in the Kandy Municipal Council (KMC), Gangawata Korale (GK), Kundasale and Gampola, and > 7 in Akurana. Majority of the families have lived in these areas for the past 26–50 years (Table 1). The monthly income category of LKR 30,000 was the highest in Akurana followed by LKR 20,000–30,000 in the other MOH areas. However, 20,000–30,000 LKR (In September 2017, 1 USD = 154.13 LKR) was identified from all other areas. According to the chi-square test for independence, there were statistically acceptable dissimilarities (*p* < 0.05) among sex ratio, average family size, number of years living in the area and monthly income of families in the entire study (Table 1).

**Table 1:** Personal and socio-demographic characteristics among study populations in the district of Kandy as a percentage (*n* = 200)

Factors		Study area					χ^2^
		Akurana	KMC	Gangawata Korale	Kundasale	Gampola	
Age	15–35	20.0^a^	39.5^b^	17.5^a^	24.5^a^	39.5^c^	χ^2^ = 87.87 (df = 8)
	35–55	45.5	41.0	59.5	50.0	56.5	
	> 55	34.5	19.5	23.0	25.5	4.0	
Sex	M	98.5^a^	89.0^b^	91.0^b^	87.5^b^	85.5^b^	χ^2^ = 28.29 (df = 4)
	F	1.5	11	9	12.5	14.5	
Family size	1 to 3	1.5^a^	16.5^b^	20.0^c^	25.5^c^	24.5^c^	χ^2^ = 94.50 (df = 8)
	4 to 6	42.0	51.5	71.5	70.0	62.0	
	> 7	56.5	32.0	8.5	4.5	13.5	
Numbers of years residing in the house	< 5	13.5^a^	7.0^b^	19.0^a^	15.0^a^	18.5^a^	χ^2^ = 46.37 (df = 12)
	6 to 25	37.5	20.0	23.0	24.0	30.0	
	26 to 50	32.0	44.0	42.5	41.5	37.0	
	> 50	17.0	29.0	15.5	19.5	14.5	
Monthly income (LKR) × 10^3^	< 5	2.0^a^	0.0^b^	0.5^c^	1.0^c^	0.0^c^	χ^2^ = 98.90 (df = 16)
	5 to 10	5.5	2.0	2.0	2.5	1.0	
	10 to 20	30.0	8.0	12.0	10.0	5.0	
	20 to 30	24.0	86.0	64.0	58.0	65.5	
	> 30	38.5	4.0	21.5	28.5	28.5	

Different superscript letters (a, b, c and d) in the first row of each factor show significant dissimilarities among the study populations (*p* < 0.05) as a post-hoc comparison as suggested by “Chi-square test for Independence”, while similar superscript letters indicate significant similarities

*KMC* Kandy Municipal Council area, *df* degree of freedom for each variable, *χ*^*2*^ Chi-square value. All contingency tests (χ^2^ tests) were significant at least at 0.05 probability level

### House condition and infrastructural characteristics

Houses having plastered cement walls with tiled or asbestos roofs were categorized as “Good”, while un-plastered brick walls with tiled, asbestos or incomplete roof (concrete slab) were considered as “Moderate”. All other types were grouped as “Poor” houses. Majority of the households in all the study MOH areas, except in Akurana, were characterized as of moderate level, while the household status of Akurana remained poor. Many of the houses were used for residential purposes, while others were used for commercial and small industrial purposes in addition to residential purposes (Table 2). Pipe borne water was the main source of water and households stored water in fully covered water tanks for usage. Households in all study areas had their toilets located separately from the house unit. However, no proper toilet facilities were detected for some houses in Akurana (3%, *n* = 200) and Kundasale (2.5%, *n* = 200). Many of the houses in Akurana were surrounded by built-up environment and marshlands, while built-up environments and agricultural areas were high in GK, Gampola and Kundasale. In the KMC, built-up environment was extremely prominent. Many of the houses were accessible through medium or small roads. All household characteristics except “type of households” and “residential function of households” were significantly different among the five MOH divisions (*p* < 0.05).

**Table 2:** Household characteristics of study areas in the district of Kandy as a percentage (*n* = 200)

Factors		Study area					χ^2^
		Akurana	KMC	Gangawata Korale	Kundasale	Gampola	
Accessibility	Main road	6.0^a^	11.0^a^	15.5^b^	4.0^a^	7.5^a^	χ^2^ = 23.82 (df = 8)
	Medium/Small road	91.0	85.5	78.5	91.0	87.5	
	Foot path/No road	3.0	3.5	6.0	5.0	5.0	
Size of the homestead (Perch)	< 5	4.0^a^	70.0^b^	2.5^a^	5.0^c^	6.0^a^	χ^2^ = 97.65 (df = 16)
	6 to 10	19.5	6.0	28.0	58.0	26.0	
	11 to 25	46.5	18.0	57.0	30.0	50.0	
	26 to 50	21.5	4.0	4.0	4.0	13.0	
	> 50	8.5	2.0	8.5	3.0	5.0	
Nature of human dwellings	Permanent	89.5^a^	99.5^b^	95.0^a^	84.5^a^	99.0^b^	χ^2^ = 54.46 (df = 4)
	Temporary	10.5	0.5	5.0	15.5	1.0	
Number of houses present in the land plot	1	72.0^a^	97.0^b^	96.0^b^	94.0^b^	96.5^b^	χ^2^ = 115.55 (df = 8)
	2 to 3	27.0	3.0	4.0	6.0	3.5	
	> 4	1.0	0.0	0.0	0.0	0.0	
Type of the households	Individual House	96.0^a^	92.0^a^	98.0^a^	96.0^a^	94.0^a^	χ^2^ = 9.10 (ns) (df = 12)
	< 5 floors	4.0	8.0	2.0	4.0	6.0	
	> 5 floors	0.0	0.0	0.0	0.0	0.0	
	other	0.0	0.0	0.0	0.0	0.0	
Residential function of the households	Residential only	93.5^a^	95.0^a^	98.0^a^	91.5^a^	95.5^a^	χ^2^ = 20.55 (ns) (df = 12)
	Residential & commercial	4.5	5.0	2.0	4.0	4.5	
	Small industry	2.0	0.0	0.0	4.5	0.0	
	Commercial only	0.0	0.0	0.0	0.0	0.0	
Status of the households	Good	29.0^a^	18.0^b^	11.5^b^	7.5^b^	16.0^b^	χ^2^ = 151.83 (df = 8)
	Moderate	24.5	66.5	75.5	70.5	66.0	
	Poor	46.5	15.5	13.0	22.0	18.0	
Number of rooms in the house	1	13.0^a^	0.0^b^	6.0^c^	4.0^c^	3.0^c^	χ^2^ = 76.09 (df = 12)
	2 to 3	76.0	95.0	94.0	94.0	92.5	
	4 to 6	10.0	5.0	0.0	2.0	3.5	
	> 6	1.0	0.0	0.0	0.0	1.0	
Vegetation coverage	Grass	39.5^a^	16.5^b^	40.5^c^	46.0^c^	56.0^c^	χ^2^ = 104.97 (df = 12)
	Bushes	26.5	48.5	45.0	41.5	47.0	
	Small trees	40.6	29.5	62.0	53.5	59.0	
	Large trees	87.5	5.5	46.5	40.5	55.0	
Surrounding landuse practices in the neighbourhood	Agricultural areas	0.0^a^	2.0^b^	16.5^c^	24.0^d^	20.0^c^	χ^2^ = 110.39 (df = 20)
	Water bodies	0.0	1.5	3.0	1.0	3.0	
	Built-up	48.0	81.5	68.5	52.0	74.0	
	Marshy	56.0	4.0	8.5	3.0	5.0	
	Abundant	12.0	5.5	5.0	24.5	7.5	
	Other	5.0	5.5	2.0	4.0	2.0	
Nature of the toilet facilities	Separate (Outside)	75.0^a^	67.0^a^	89.5^b^	88.5^b^	79.5^a^	χ^2^ = 81.16 (df = 12)
	Attached	40.0	50.0	23.0	15.5	41.0	
	Both	18.0	17.0	12.5	8.5	20.5	
	None	3.0	0.0	0.0	2.5	0.0	
Water source	Well	2.0^a^	0.0^a^	0.0^a^	0.0^b^	2.0^a^	χ^2^ = 38.98 (df = 12)
	Tube-well	4.0	0.0	6.0	10.0	3.0	
	Pipe	98.0	100.0	100.0	100.0	99.0	
	Other	0.0	0.0	0.0	0.0	0.5	
Roofing conditions	Concrete	42.0^a^	38.5^a^	24.0^b^	10.5^c^	30.0^b^	χ^2^ = 109.93 (df = 16)
	Roof tiles	31.0	21.0	56.0	37.5	50.5	
	Asbestos	53.0	63.5	71.0	78.0	71.0	
	Metal sheets	61.0	53.0	27.5	68.0	31.5	
	Other	3.0	2.5	4.0	10.5	4.5	

Different superscript letters (a, b, c and d) in the first row of each factor show significant dissimilarities among the study populations (*p* < 0.05) as a post-hoc comparison as suggested by “Chi-square test for Independence”, while similar superscript letters indicate significant similarities

*KMC* Kandy Municipal Council area, *df* degree of freedom for each variable, *χ*^*2*^ Chi-square value. Except for “type of households” and “residential function of households”, all contingency tests (χ^2^ tests) were significant at least at 0.05 probability level. ns – not significant at 0.05 probability level

### Knowledge attitude practices (KAPs)

Knowledge, Attitude and Practices related parameters of the study populations are given in Table 3. The state of awareness was High (Good) in four MOH areas with nearly 40% or greater except in Kundasele, which was significantly less. Considering good and medium levels of awareness as satisfactory, only the KMC and Kundasale had unsatisfactory levels at 23%. During the study it was noted that the residents had limited knowledge of morphological features, life cycle, active time of dengue vectors and role of the community participation for dengue prevention/control. However, study community was well aware of the names of the major vectors of dengue, preventive measures against dengue and diverse nature of breeding habitats.

**Table 3:** Knowledge Attitude Practices (KAP) among study populations on dengue in Kandy district as a percentage (*n* = 200)

Factors		Study area					χ^2^
		Akurana	KMC	Gangawata Korale	Kundasale	Gampola	
Knowledge							
Awareness about dengue	Good	39.5^a^	40.0^a^	41.5^a^	25.5^b^	47.5^a^	χ^2^ = 38.12 (df = 8)
	Medium	44.5	37.0	47.5	51.0	42.5	
	Poor	16.0	23.0	11.0	23.5	10.0	
Attitudes							
Nature of case frequency in the area	Frequent	56.0^a^	53.5^a^	53.0^a^	35.5^b^	29.5^b^	χ^2^ = 48.37 (df = 8)
	Occasionally	43.0	45.5	46.5	62.5	69.0	
	None	1.0	1.0	0.5	2.0	1.5	
Need for better awareness and knowledge on dengue	Yes	48.0^a^	58.0^a^	60.0^a^	61.5^a^	84.0^b^	χ^2^ = 59.59 (df = 4)
	No	52.0	42.0	40.0	38.5	16.0	
If yes, in which aspects	Symptoms and treatments of DHF	75.0^a^	64.0^a^	58.5^b^	64.8^b^	78.0^a^	χ^2^ = 49.75 (df = 8)
	Controlling of vectors	53.5	42.0	69.5	71.5	89.0	
	SWM	45.0	58.0	34.0	29.5	57.0	
Willingness to contribute community based controlling activities	Yes	84.5^a^	59.5^b^	76.0^c^	71.5^c^	87.0^a^	χ^2^ = 52.76 (df = 4)
	No	15.5	40.5	24.0	28.5	13.0	
Reasons for not practicing of composting or home gardening, due to restrictions in	Time	52.0^a^	68.0^b^	42.5^c^	39.5^c^	34.0^d^	χ^2^ = 64.83 (df = 8)
	Space	62.0	78.0	52.0	42.0	52.0	
	Labour	12.0	54.0	21.5	18.0	49.0	
Practices							
Cleanliness of the homestead	Yes	92.0^a^	78.0^b^	96.0^a^	95.0^a^	94.5^a^	χ^2^ = 55.05 (df = 4)
	No	8.0	22.0	4.0	5.0	5.5	
Practice of composting or home gardening	Yes	18.0^a^	2.5^a^	24.5^a^	18.5^a^	25.5^a^	χ^2^ = 18.31 (df = 4)
	No	82.0	97.5	75.5	81.5	74.5	
Waste disposal frequency	Daily	21.0^a^	6.5^b^	16.5^c^	8.0^b^	14.5^c^	χ^2^ = 71.79 (df = 8)
	< 7 Days	73.5	93.5	83.5	92.0	85.5	
	> 7 Days	5.5	0.0	0.0	0.0	0.0	
Waste disposal method	Garbage pit	28.0^a^	4.0^b^	15.0^c^	10.5^c^	18.5^d^	χ^2^ = 142.46 (df = 20)
	Collected by the Municipality	58.0	98.0	79.5	69.0	73.0	
	To road	0.0	0.0	0.0	0.0	0.0	
	Open ground	0.0	0.0	0.0	0.0	0.0	
	Composting	2.0	4.0	8.5	15.0	18.0	
	Burning	34.5	12.0	38.0	44.0	34.0	
Nature of the waste collection Service of Pradeshiya Sabha or Municipality	Once 2 week	0.0^a^	1.5^a^	2.0^b^	0.0^b^	0.0^a^	χ^2^ = 94.54 (df = 20)
	Once a week	74.0	94.0	78.0	79.0	83.0	
	Irregular	83.5	46.0	65.0	59.5	51.5	
	Use no alarming sound	32.0	6.0	44.0	38.0	12.0	
	Don’t reach the road or house	53.0	0.5	37.5	27.0	11.0	
	Item rejection	84.0	88.0	87.0	83.0	89.0	
Source separation of Solid Waste	Yes	37.5^a^	35.5^b^	20.5^a^	18.5^a^	32.5^c^	χ^2^ = 30.10 (df = 4)
	No	62.5	64.5	79.5	81.5	67.5	
Used Mosquito bite prevention method	Screen	23.5^a^	39.0^c^	14.5^b^	10.5^b^	28.5^a^	χ^2^ = 113.01 (df = 24)
	Close windows	39.0	17.5	42.0	38.0	25.5	
	Coils/use of smoke	69.0	81.0	62.5	70.5	78.5	
	Nets	54.5	52.0	68.5	63.5	75.5	
	Fans	27.0	49.0	12.5	14.0	30.5	
	Other	42.5	7.5	28.5	32.0	40.0	
	None	4.5	0.0	2.0	1.5	0.0	
Covering status of the water storage tanks	Fully covered	80.5^a^	93.0^b^	96.5^b^	85.0^c^	99.0^d^	χ^2^ = 89.77 (df = 8)
	Partially covered	1.5	0.0	0.5	7.0	1.0	
	None	18.0	7.0	3.0	8.0	0.0	
Status of Roof Gutters	Functioning	48.0^a^	58.0^a^	35.0^b^	37.5^b^	29.5^b^	χ^2^ = 55.52 (df = 8)
	Blocked	2.0	1.0	0.0	0.0	0.0	
	None	50.0	41.0	65.0	62.5	70.5	

Different superscript letters (a, b, c and d) in the first row of each factor show significant dissimilarities among the study populations (*p* < 0.05) as a post-hoc comparison as suggested by “Chi-square test for Independence”, while similar superscript letters indicate significant similarities

*KMC* Kandy Municipal Council area, *df* degree of freedom for each variable, *χ*^*2*^ Chi-square value. All contingency tests (χ^2^ tests) were significant at least at 0.05 probability level

In case of attitudes, a high proportion of individuals was willing to improve their awareness on the aspects of controlling dengue vector breeding, solid waste management (SWM), and recognizing symptoms and treatment strategies of dengue. Majority of individuals among the study populations were willing to extend their support in controlling dengue through supporting community based controlling strategies.

The cleanliness of the homesteads was high among all study populations. Although most of the households did not practice composting or home gardening, the proportion of such households were comparatively higher in GK and Gampola. Limitations posed by space and time were indicated as reasons for not following such practices. Even though the majority of households did not practice source separation of solid waste, a higher proportion in Akurana, Gampola and KMC did so. Waste collection by the local authorities and open burning were observed as the main waste disposal practice. Although the frequency of waste disposal in general was < 7 days, the selected people in Akurana area indicated the removal of waste as a daily practice (23%, *n* = 200) compared to other areas. Use of bed-nets, mosquito coils, and creation of smoke were common practices to avoid mosquito biting. According to chi-square test of independence, it was evident that there were significant differences of KAP among the five populations studied in the Kandy district (*p* < 0.05).

### Service provided by the health authorities for vector control

Majority of the individuals indicated that there were no projects conducted to control dengue in their respective areas, except for Akurana. A notable proportion (35.0%, *n* = 200) in the KMC responded that projects had been conducted earlier. Almost all of the vector control activities such as volunteer cleaning programmes (*Shramadaana*) and awareness programmes etc. were conducted by the private sector with the support of government and Non-Government Organizations (NGOs). In certain localities (especially in Akurana), the religious leaders emerged as one of the key community mobilization agents that motivated and encouraged the local communities towards controlling of dengue vectors, via raising awareness and instructing to organize *Shramadaana*. Overall, the study communities were not satisfied with the services provided by health authorities for vector control in their areas. Chi-square test of independence also showed that there were differences between the 5 population in terms of roles of vector control entities (*p* < 0.05; Table 4).

**Table 4:** Role of Vector Control Entities (VCE) in respective study areas in the district of Kandy Kandy as a percentage (*n* = 200)

Factors		Study area					χ^2^
		Akurana	KMC	Gangawata Korale	Kundasale	Gampola	
Has there been any project to control dengue	Yes	64.5^a^	35.0^b^	15.0^c^	12.0^c^	18.0^c^	χ^2^ = 85.15 (df = 4)
	No	35.5	65.0	85.0	88.0	82.0	
If Yes, the implementing party	Government	5.0^a^	18.0^b^	3.5^a^	2.0^a^	7.0^a^	χ^2^ = 53.89 (df = 8)
	Non-Government Organizations (NGO)	1.0	2.0	0.0	1.0	1.0	
	Private Sector	94.0	80.0	96.5	97.0	92.0	
Attitudes on the role of the PHI	Excellent	29.5^a^	38.0^a^	25.0^a^	22.0^b^	31.0^a^	χ^2^ = 76.80 (df = 12)
	Visits when there is an issue only	43.5	34.0	38.0	20.4	34.0	
	Have to meet when there is an issue	2.0	3.5	5.0	14.1	11.0	
	Has not influenced any matter on dengue	25.0	24.5	32.0	43.5	24.0	

Different superscript letters (a, b, c and e) in the first row of each factor show significant dissimilarities among the study populations (*p* < 0.05) as a post-hoc comparison as suggested by “Chi-square test for Independence”, while similar superscript letters indicate significant similarities

*KMC* Kandy Municipal Council area, *df* degree of freedom for each variable, *χ*^*2*^ Chi-square value. All contingency tests (χ^2^ tests) were significant at least at 0.05 probability level

### Euclidean distance based clustering

**Fig. 2:**
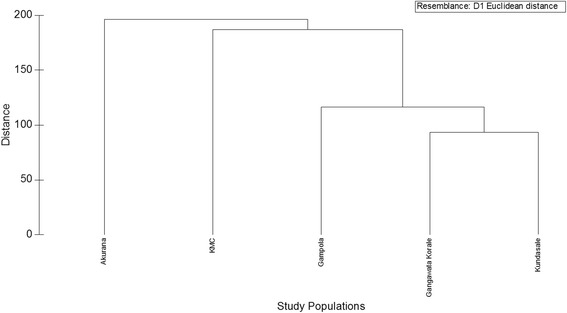
Dendrogram of the cluster analysis of the five study populations in terms of the studied demographic, epidemiological and socio-economic characteristics, showing three clusters at about 120 dissimilarity level of Euclidean distance

**Fig. 3:**
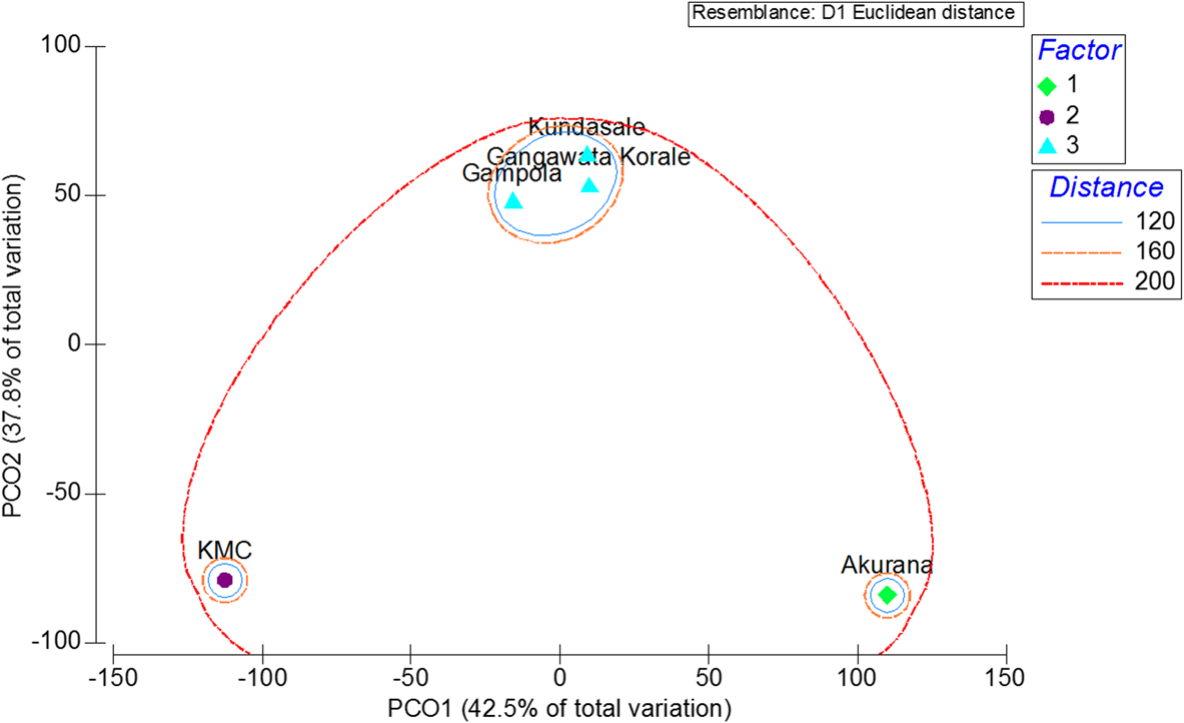
Two dimensional (PC_1_ and PC_2_) ordination PCO analysis of the populations in the study MOHs in terms of demographic, KAPs, socio-economic and household related characteristics

The status of clustering in the study populations with respect to demographic, KAPs, socio-economic and household related characteristics is illustrated in Fig. 2. According to the Euclidean distance, at about 120 dissimilarity level, KMC and Akurana are highly dissimilar from the other three populations. The levels of dissimilarity between GK, Gampola and Kundasale were comparatively low. From this analysis, the emergence of three clusters for KMC, Akurana and other MOH areas can be recognized (Fig. 2). The Global R value of 0.98 and pair-wise comparison from the Analysis of Similarities (ANOSIM) also confirmed that the demographic, KAPs, socio-economic and household related characteristics among the three clusters resulted in cluster analysis were significantly different (*p* < 0.05) from each other. Further, a high cumulative percentage (85.4%) of the total variation among the study populations was accounted for by first two axes (PC_1_ and PC_2_) of the Principal Coordinates (PCO), confirming the sufficiency of two-dimensional ordination of the five populations. These ordinations also indicated that the five populations represented three clusters, which further confirmed the clustering at the Euclidean of distance of 120 (Fig. 3).

## Discussion

Vector-borne diseases, especially by mosquitoes, are recognized as a major health issue in many tropical countries, due to the alarmingly increasing number of people affected and their geographical spread. Similar to other community health issues, knowledge attitudes and practices (KAPs), epidemiological, socio-economic and demographic factors of the population play a critical role in both incidence of dengue epidemics and implementation of control measures [8, 9]. Therefore, this study was conducted to characterize the demographic, household related, socio-economic and KAPs in five selected dengue free communities living in dengue risk areas within the Kandy District, which is considered as the fourth high risk dengue district in Sri Lanka [6].

### Demographic factors

Demographic factors such as gender, age and family size influence the risk of dengue transmission [8, 17–19]. In accordance with traditional customs, the males are considered as household heads in all the communities in this study. Of the study areas, Kandy Municipal Council (KMC) is a highly urbanized area, while both Gampola and Akurana are semi-urban areas. Kundasale and Gangawata Korale (GK) are characterized by rural conditions. All the study communities belonged predominantly to the Sinhalese ethnic group, except Akurana where Muslims were predominant.

The age group of 35–55 years dominated all the study populations, while the age group of 15–35 years was the least frequent group. However, a previous study carried out in the Kelaniya Medical Officer of Health (MOH) area in Sri Lanka, has indicated that the age group of 6–18 years as the vulnerable age range for the infection of dengue followed by 19–55, 1–5 and > 55 years [8]. The education level, family income and KAPs of the families may have played a major role in maintaining the dengue free status of the study population [17, 19]. The presence of a high number of family members in the households apparently imposes a high responsibility on the parents or guardians encouraging them to maintain a clean and safe environment to prevent their family becoming ill [17]. The average family size was 4–6 members in all study communities, except in Akurana, where it was > 7 individuals.

Families with a moderate income were dominant in all the study populations (except in Akurana). Usually, middle class families with a moderate to high educational background are known to be more dedicated towards the cleanliness of the environment and the health of their family members thereby reducing the breeding of *Aedes* vectors [18, 20–22]. As suggested by previous records on dengue patients, the highest number of dengue cases was always reported from the KMC, which is characterized by urbanization and a higher population density than rest of the study areas. Some studies carried out in Sri Lanka and Malaysia, have also made similar observations, indicating that urbanization could impose a high vulnerability on the incidence of dengue epidemics [8, 23].

### Household related characteristics

Features and characteristics of the living environment such as nature of the house, roofing conditions, water supply and storage facilities, surrounding vegetation and land use practices directly affects the breeding of *Aedes* vectors, thereby influencing the incidence of dengue epidemics [8, 20, 22–24]. Approximately, more than 84% of the households had individual permanent houses in moderate condition (> 66%) that were utilized for residence in all the study MOH areas. There is evidence that the occupants of moderate or smaller households had a relatively higher likelihood of maintaining a cleaner environment and of taking adequate measures to control vectors in contrast to families living in relatively larger households [17, 18]. The present study denoted an average of 2–3 rooms per house in all study areas which are considerably moderate in size. Therefore, absence of dengue infections among the target population in the present study may also advocate the above finding.

Concrete, asbestos and metal sheets were the preferred methods of roofing in Akurana and KMC, while roof tiles and asbestos were the preferred materials in GK, Gampola and Kundasale. Even though concrete slabs and stagnant drains or gutters with fallen leaves and other debris could act as ideal breeding places for *Aedes* mosquitoes, especially during the rainy season [23, 24]. However, proper attention and maintenance of a cleaner environment within the households have ensured the dengue free status within the study population in the present study.

Availability of pipe borne regular water supply (provided by the National Water Supply and Drainage Board) was the major water source in all the study areas. This has encouraged residents to maintain a smaller number of large water containers for the storage of water [25, 26]. This is different from many Asian countries such as Malaysia and Indonesia [20, 23, 25]. Furthermore, residents have been encouraged to use fully covered water tanks to cater for their water storage. While open water storage containers provide ideal breeding places for dengue vectors, the utilization of fully covered water containers may be contributing for controlling of *Aedes* mosquitoes [20, 25, 26].

The average plot size of the homesteads in Akurana, Gampola and GK was larger in extent (11–25 perch), when compared to that of KMC. The highly urbanized nature of the KMC may have contributed to the reduced land availability resulting in conjugation of families. Comparatively, a high proportion (27.0%, *n* = 54) of land plots in Akurana had 2–3 houses within a single land plot. This is due to socio-economic reasons and cultural traditions that result in related families living close to their origin with strong interactions as a large unit. However, high number of family units living in the same land plot with a high degree of responsibility and strong interactions will entrust a high degree responsibility on the elders of the family to take care of their welfare. This has ensured that they maintain a clean and mosquito free environment for the well-being of their families. Further, the availability of human resources may also have ensured the maintenance of a clean environment within the land plot minimizing the risk of dengue incidence [17].

Small and large trees were the common vegetation types in all the study areas except in the KMC. This may also be a contributing factor for the dengue free status of the study populations, since *Aedes* species have been found to prefer less vegetation [23]. Built-up environments were identified as the predominant surrounding types of the households in all the study areas along with agricultural and marshy lands as other land use types, except in KMC. However, a recent study has also observed that built-up environments are the dominant land use type in a randomly selected cluster of dengue patients in the Kelaniya MOH area in the Gampaha District in Sri Lanka [8]. Therefore, maintenance of a cleaner environment around the households may be the reason for dengue free status within the current study communities.

### Knowledge attitude practices (KAP)

The Knowledge Attitude Practices of a community play a major role in incidence of dengue epidemics [17, 18] and controlling of dengue through community involvement [17, 20, 25]. The cleanliness of the homesteads was high in all study MOH areas, while the cleanliness in the KMC was comparatively lower than the rest. The awareness levels on dengue in the study communities were high to moderate; this may have directly contributed to their clean and dengue free status even while living in a high dengue risk area [17, 18]. The study communities had a limited knowledge on morphological features, life cycle, active time of dengue vectors and role of the community participation for dengue prevention/control. A study carried out in Jeddah located in Saudi Arabia, has noted that more than 50% of the participants lacked the knowledge on vector morphology and active time of dengue vectors, similar to the findings of current study [29].

However, the current study indicated that the community possessed a fair knowledge on preventive measures against dengue and diverse nature of breeding habitats that may contribute to their dengue free status, regardless of residing in high dengue risk areas. Several studies carried out in India [27], Brazil [28] and Saudi Arabia [29], have reported fairly higher knowledge rate in comparison to the present study population. Therefore, proper knowledge on the morphology and behaviour of dengue vectors could be important in maintaining the healthy status of the community.

A high proportion of individuals was willing to improve their awareness on the aspects of controlling dengue vectors breeding, solid waste management (SWM), symptoms and treatment strategies of dengue. Consciousness on signs, symptoms and treatment strategies of dengue would be crucial in recognizing the disease and in obtaining timely medical care, which would reduce the number of deaths. Improving the awareness on such aspects have been recommended by several studies carried out at local and international scales in implementing successful dengue controlling programmes via community participation [17, 20, 23, 30]. Majority of individuals in all MOH areas expressed their willingness to support dengue control through community based controlling strategies. Hence, the relevant Vector Control Entities could cooperate with the local community in designing community based vector controlling strategies [20, 25].

Waste collection by the Municipal Council (MC) and open burning were observed as the predominant waste disposal practices, while the frequency of waste disposal was < 7 days. A high proportion of households (23.0%, *n* = 46) in Akurana practiced daily disposal of waste. Some households in Akurana, Gampola and KMC practiced source separation of waste. Maintaining solid waste for a long time, often more than seven days, has been found to enhance the breeding of *Aedes* mosquitoes and thereby increase the transmission of dengue. Thus, the proper disposal of solid waste by the local authorities and source separation of solid waste could contribute to reducing the transmission of dengue, within the studied families [20, 25].

Irregular frequency of waste collection, avoiding or not visiting some households/roads, and rejection of certain items during collection were some drawbacks in the waste collection services of KMC. Hence, these limitations should be addressed by the relevant authorities to provide a satisfactory waste collection service, which was a critical requirement for management of dengue epidemics in the study areas. A community involved intervention could be of use in designing such programmes that has often proved fruitful in controlling dengue [20, 25]. In addition, most of the houses in all MOHs did not have gutters and existing gutters were properly maintained. The awareness programmes that were aired through television during the severe epidemics in 2009, are attributed to such practices that have restricted the formation of ideal breeding habitats for *Aedes* mosquitoes [25, 30]. The present study recognized the use of bed-nets, mosquito coils, fans, screens and creation of smoke as the common practices to avoid mosquito bites. A study conducted within the Kelaniya MOH area, also indicated that use of bed-nets, mosquito coils and screens are the preferred mosquito bite prevention methods among the public [8].

### Role of vector control entities (VCE)

The role of the Vector Control Entities (VCE) at local level has been questioned by the majority of the study communites (except in KMC and Kundasale), suggesting that they are not contented with the contribution of VCEs. The findings of Nazeer and de Silva [30] indicated that the government, NGOs and media are making significant efforts to raise awareness of the people on dengue to achieve controlling of dengue vectors and these programmes are often effective and successful [30]. Yet, majority of the individuals indicated that there have been no projects conducted to control dengue in their respective areas, except for Akurana. Tendency of the relevant VCEs to fully operate only within urban areas, while limiting their activities to general entomological inspections within the semi-urban and rural areas at which the current study was conducted, may be the reason for such observation. Hence, it is important to point out that the general community is expecting more effort and contribution from relevant entities (government, NGOs and volunteers) in providing the required knowledge and resources for control of dengue. A recent study has also highlighted similar trends among the community in Jamaica, emphasizing importance of the role of government entities in controlling of dengue [17]. It must be noted that the initiatives of NGOs were predominantly organized and motivated by religious leaders in the area. For example, in Akurana, it was observed that religious leaders play a vital role in encouraging local community for vector control through volunteer cleaning programmes (*Shramadaana*), working in cooperation with government entities and raising awareness of the disease.

A study conducted in Thailand to determine the pathway of dengue transmission within a population by using geolocated genotype (*n* = 88) and serotype data (*n* = 17,291), has found that 60% of the dengue cases who were living < 200 m apart came from the same transmission chain, bearing evidence of the sequential transmission of the virus between households in densely populated locations [31]. This study underlines the significance of the immediate environment in the origin/spread of the disease and necessity of targeted vector control in the vicinity of detected cases. Therefore, the present study caters to this requirement by identifying the critical aspects of the community for driving of integrated vector management activities, through a systematic study of KAPs, socio-economic, household related and demographic characteristics of dengue free individuals residing in high risk areas. Further, current study attempts to widen the knowledge on such aspects, so that government entities, NGOs, religious and political leaders could focus on such aspects in motivating the community to cooperate in integrated vector management strategies to ensure success and sustainability in controlling dengue vectors [20, 25]. The findings of the present study contribute to designed and improve vector control programmes in order to diminish dengue transmission within the Kandy District, Sri Lanka.

## Conclusions and recommendations

As suggested by the findings, knowledge and awareness regarding dengue, especially in prevention of vector breeding, bites of mosquitoes, symptoms and patient management, and SWM should be improved. The attitudes of the community towards home gardening, composting, SWM, maintenance of a clean and dengue free environment and community participation for vector controlling activities should be enhanced. The role played by the religious leaders, especially in Akurana, was found to be a promising approach to enhance community based dengue vector management strategies by guiding the local community. Furthermore, government agencies have to reassess the efficiency of or revamp several critical services such as garbage collection and services of VCE at the local level.

## Additional file

Additional file 1: Questionnaire for Household Survey (The questionnaire used to collect the relevant information of household heads in the study population approved by the Ethics Review Committee, Faculty of Medicine, University of Kelaniya). (DOCX 60 kb)

## Abbreviations

GK: Gangawata Korale
GND: Grama Niladhari Division
KAP: Knowledge Attitude Practices
KMC: Kandy Municipal Council
MC: Municipal Council
MOH: Medical Officer of Health
NGOs: Non-Government Organizations
PHI: Public Health Inspector
SWM: Solid Waste Management
VCE: Vector Control Entities
