# Supplementary material for: Socio-economic, Knowledge Attitude Practices (KAP), household related and demographic based appearance of non-dengue infected individuals in high dengue risk areas of Kandy District, Sri Lanka
Source: BMC Infect Dis. 2018 Feb 21;18:88. doi: 10.1186/s12879-018-2995-y (PMC5822474; doi:10.1186/s12879-018-2995-y)
Supplement: Supplementary file 1 — Questionnaire for Household Survey (The questionnaire used to collect the relevant information of household heads in the study population approved by the Ethics Review Committee, Faculty of Medicine, University of Kelaniya). (DOCX 60 kb) [file 12879_2018_2995_MOESM1_ESM.docx]

**
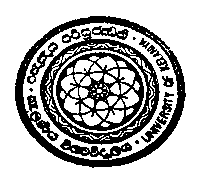
**

**FACULTY OF MEDICINE**

**UNIVERSITY OF KELANIYA, SRI LANKA**

**P.O. Box 6, Thalagolla Road, Ragama**

**Questionnaire for Household Survey**

**Project Title:** Comparative characterization of risk factors and risk mapping of dengue transmission in Colombo and Kandy districts of Sri Lanka.

1. Interview date: …………………………………………………………………………...
2. Ref No: ……….…………………………….……………………………………………
3. Site ID: …………………………………….……………………………………………..
4. MOH area: …………………………………………………………………………….....
5. PHI area: ………………………………………………………………………………....
6. GN division: ……………………………………………………………………………..
7. House ID: ……………………….……………………….……………………………….
8. GPS location: ……………………………………………………………………………
9. Address: ………………………..…………….………………………………………….

……………………………………………………………………………………………

1. Name of the household head: …………………………………………………..

...………………………………………………………………………………………

1. Gender of the household head: …………………………………………………
2. National Identity Card number: ………………………………………………………….
3. Size of the homestead (perches): …………………………………………………………
4. How long have you been living here (in years)?.................................................................
5. Number of persons in household:…………………………………………………………
6. Accessibility: Main road Small No road
7. Household details:

| No. | Name of the member | Relationship | Sex | Age | Educational Status | Employment/ If yes, occupation and official address | Monthly income | Dengue Status (Y/N) |
| --- | --- | --- | --- | --- | --- | --- | --- | --- |
| 1. |  |  |  |  |  |  |  |  |
| 2. |  |  |  |  |  |  |  |  |
| 3. |  |  |  |  |  |  |  |  |
| 4. |  |  |  |  |  |  |  |  |

| - <5000 |  |
| --- | --- |
| - 5001-10000 |  |
| - 10001-20000 |  |
| - 20001-30000 |  |
| - 30000< |  |

1. Total family income level: (Please tick √)
2. Number of houses within the land plot: ……………………………………………….
3. No. of bedrooms in the house:…………………………………………..
4. Status of the premises: (Please tick √)

| - Low |  |
| --- | --- |
| - Moderate |  |
| - High   ………………………………………………… |  |
| - Other |  |

| - Permanent |  |
| --- | --- |
| - Temporary |  |

1. Human dwellings:
2. Type of houses : (Please tick √)

| - Mainly individual houses (detached or semi-detached) |  |
| --- | --- |
| - Mainly apartment building with less than 5 floors |  |
| - Mainly apartment building with 5 or more floors |  |
| - Other   …………………………………………………………………………………………….. |  |

1. Residential function (Please tick √)

| - Residential only |  |
| --- | --- |
| - Residential and commercial |  |
| - Residential with small private industry |  |
| - Mainly commercial with few residences |  |
| - Other…………………………………………... … |  |

1. Surrounding cleanliness: (Please tick √ your answer)

| - Yes |  |
| --- | --- |
| - No |  |

1. Surrounding: (Please tick √)

| - Agricultural areas |  |
| --- | --- |
| - Water bodies |  |
| - Built-up Environment |  |
| - Marshy lands |  |
| - Other ……………………….. |  |

1. Awareness and Knowledge about dengue (Use the supplementary questionnaire:

Good Poor Nil

1. Vegetation coverage: (Please tick √)

| - Grass |  |
| --- | --- |
| - Bushes |  |
| - Small trees |  |
| - Large trees |  |

1. Sanitation & waste disposal frequency: Daily Within 7 days >7 days

| - Burring in to the garbage pit |  |
| --- | --- |
| - Collecting tailors of Municipal Council |  |
| - Discarding to the road |  |
| - Open ground |  |
| - Composting |  |
| - Collecting and burning |  |
| - Other   ……………………………………………………………… |  |

1. Waste disposal method: (Please tick √)
2. How do you assess the service provided by the Praddeshiya Sabha (PS)/ Urban Council (US) to collect solid waste that you have collected at household level? (Please tick √)

| - They frequently send their tractors to collect solid wastes |  |
| --- | --- |
| - Tractor comes once in every two weeks |  |
| - Tractor comes once in every month |  |
| - Their service is irregular |  |
| - Never coming |  |
| - Other (specify)   …………………………………………………………………… |  |

1. Are there any bottlenecks or gaps in the waste collection service of the PS/UC? (Please tick √)

| - They don’t mind the date/week they are coming |  |
| --- | --- |
| - They don’t display the sound to aware us |  |
| - They don’t reach our houses/road |  |
| - They don’t accept some items such as door glasses |  |
| - They expect tips from us for their service |  |
| - Other (specify)   …………………………………………………………………… |  |

1. Do you practice home gardening and composting at your homestead? (Please tick √)

| - Yes |  |
| --- | --- |
| - No |  |

1. If “not”, what are the reasons?

…………………………………………………………....………………………………………………………………………………............................................................................

1. Use of mosquito control measures:

| - Yes |  |
| --- | --- |
| - No |  |
| - Screens |  |
| - Insecticides |  |
| - Close windows |  |
| - Smoke/ burning coils |  |
| - Mosquito net |  |
| - Other (specify) |  |

1. Toilet condition:

| - Available, Outside |  |
| --- | --- |
| - Non Available |  |
| - Normal |  |
| - Attached bathroom/s |  |

1. Roofing condition:

| - Asbestos sheets |  |
| --- | --- |
| - Roof tiles |  |
| - Metal sheets |  |
| - Other ……………………………………….. |  |

1. Gutter condition:

| - Available |  |
| --- | --- |
| - Non Available |  |
| - Properly Functioning |  |
| - Non functioning |  |

| - Ground-well water |  |
| --- | --- |
| - Tube-well water |  |
| - Piped water |  |
| - Other   ……………………………………………….………… |  |

1. What is your source of water? (Please tick √)
2. Protection of water storage container

| - Fully covered |  |
| --- | --- |
| - Partially covered |  |
| - Sometimes covered |  |
| - Not covered |  |

1. Potential breeding sites for *Aedes* larvae: Yes No
2. Entomological findings:

1 : *Ae. Aegypti* 2: *Ae. Albopictus*

| Type | 1 | | | 2 | | | 3 | | | 4 | | |
| --- | --- | --- | --- | --- | --- | --- | --- | --- | --- | --- | --- | --- |
|  | Source | Species | | Source | Species | | Source | Species | | Source | Species | |
|  |  | 1 | 2 |  | 1 | 2 |  | 1 | 2 |  | 1 | 2 |
| Larvae |  |  |  |  |  |  |  |  |  |  |  |  |
| Pupa |  |  |  |  |  |  |  |  |  |  |  |  |
| Adult |  |  |  |  |  |  |  |  |  |  |  |  |

1. Has there been any project to control dengue in your area before this project? (Please tick √)

| - Yes |  |
| --- | --- |
| - No |  |
| - Do not know |  |

1. If yes, who implemented it?...........................................................................................

| - Excellent |  |
| --- | --- |
| - Good |  |
| - Satisfactory |  |
| - Not satisfactory |  |
| - Highly not satisfactory |  |

1. How do you assess it? (Please tick √)
2. If failed, what are the reasons?

- …………………………………………………………………………
- …………………………………………………………………………
- …………………………………………………………………………
- …………………………………………………………………………

1. Have you experienced many mosquito related health issues before introduced the current project? (Please tick √)

| - Yes, frequently |  |
| --- | --- |
| - Yes, occasionally |  |
| - No, any case reported/known |  |

1. Are you using separate bags for collection of polythene, glasses, and papers prior disposal? (Please tick √)

| - Yes |  |
| --- | --- |
| - No |  |

1. Have you ever taken awareness about Dengue viral fever and how it can be controlled before this project? (Please tick √)

| - Yes |  |
| --- | --- |
| - No |  |

1. Who gave such awareness and when it happened?

| Year | Conducted by |
| --- | --- |
|  |  |
|  |  |
|  |  |

| - VCEs have done a good job |  |
| --- | --- |
| - VCEs have not influenced us in these matters |  |
| - VCEs visit us when there is an issue only |  |
| - We had to go and meet VCEs, when there is an issue |  |
| - Other (specify)………………………………………... |  |

1. In terms of mosquito control and waste management, how do you assess role of the Vector Controlling Entities (VCE) in your area before this project? (Please tick √)
2. According to your vision, what are the suggestions of you to improve this project?

1. ……………………………………………………………..

2. ……………………………………………………………..

1. Do you expect some more awareness activities related to dengue fever and prevention of dengue mosquito? (Please tick √)

| - Yes |  |
| --- | --- |
| - No |  |

1. If ‘yes’ what the areas need to be paid a special attention? (Please tick √)

| - Recognition of symptoms and treatments for dengue fever |  |
| --- | --- |
| - Solid waste management and the environment |  |
| - Prevention of dengue mosquito breeding and biting |  |
| - Other (specify) …………………………………………… |  |

| - Yes |  |
| --- | --- |
| - No |  |
| - Do not know |  |

1. Do you think that this project will support you to control mosquito related issues in your area? (Please tick √)
2. If “yes” how it will be successful? (Please tick √ your answers)

| - This is a well-organized project |  |
| --- | --- |
| - It meets our aspirations and willingness |  |
| - It gets maximum community support |  |
| - The research team urges us through awareness |  |
| - Other (specify)   ………………………………………………………………… |  |

1. If “not”, give reasons

………………………………………………………………………………………………………………………………………………………………………………………………

1. What is your overall idea about this Dengue Controlling Project, conducted by the Faculty of Medicine, University of Kelaniya? (Please tick √)

| - Excellent |  |
| --- | --- |
| - Good |  |
| - Satisfactory |  |
| - Not satisfactory |  |
| - Highly not satisfactory |  |

1. If the University of Kelaniya wishes to implement a dengue control program in your area, do you wish to support it? (Please tick √)

| - Yes |  |
| --- | --- |
| - No |  |

**Special notes:**

……………………………………………………………………………

……………………………………………………………………………

…………………… ………………………...

Date Signature of the household head
